# Supplementary material for: The Cross-Resistance Pattern and the Metabolic Resistance Mechanism of Acetamiprid in the Brown Planthopper, Nilaparvata lugens (Stål)
Source: Int J Mol Sci. 2022 Aug 21;23(16):9429. doi: 10.3390/ijms23169429 (PMC9409256; doi:10.3390/ijms23169429)
Supplement: Supplementary file 1 [file ijms-23-09429-s001.zip › ijms-1855326-supplementary.pdf]

Supplementary Information

Supplementary Figures

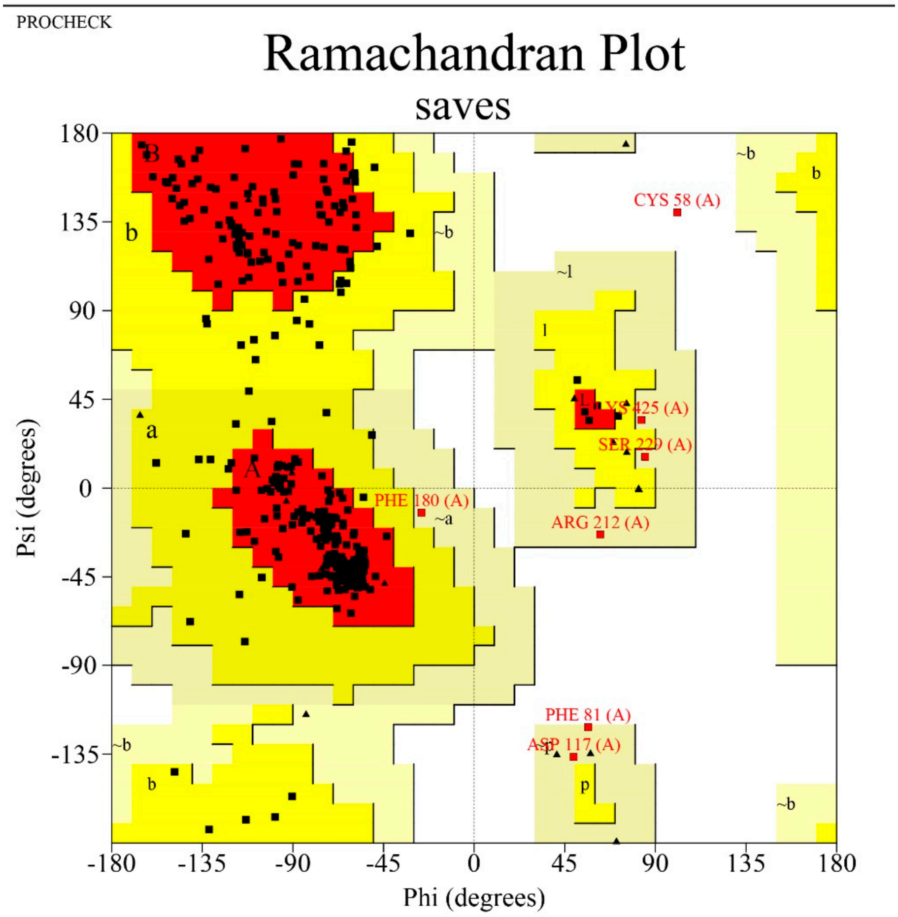

Plot statistics

Residues in most favoured regions [A,B,L]	317	86.6%
Residues in additional allowed regions [a,b,l,p]	42	11.5%
Residues in generously allowed regions [~a,~b,~l,~p]	6	1.6%
Residues in disallowed regions	1	0.3%
	----	------
Number of non-glycine and non-proline residues	366	100.0%
Number of end-residues (excl. Gly and Pro)	2	
Number of glycine residues (shown as triangles)	21	
Number of proline residues	26	

Total number of residues	415	

Based on an analysis of 118 structures of resolution of at least 2.0 Angstroms and R-factor no greater than 20%, a good quality model would be expected to have over 90% in the most favoured regions.

Supplementary Figure. S1. Evaluation of homologous modeling of CYP6ER1 monooxygenase of *N. lugens*.

Note: Generally, the proportion of amino acid residues lying in the most favoured allowed regions and the additional allowed regions in the whole protein is higher than 90%, and the number of amino acids lying in the disallowed region should be less than 5% of the total number of amino acids. We can conclude that the conformation of the model conforms to the rules of stereochemistry.

Supplementary Table S1: Primers used in qRT-PCR and dsRNA synthesis

	Gene name	Forward primer (5′-3′)	Reverse primer (5′-3′)
CYP4 Clade	*CYP4C62*	CAACTGCTGGTATCTGCTGGTCTC	TTCATAACACACCCTGCAGGAAC
	CYP4C61	CAGCTGGTGTTTGGGTCAACATC	CAGATATCCTCTTGCTTTTCAGTC
	CYP4C76	GCAATGGTCTGGCCCATTTGCAG	GAAACCTACTCTTCTCACCAAATGT
	CYP4C77	TGTTGAGATTATAGCCAGCAGTG	GTGTGAGAAACGGGTAGATATCG
	CYP4C78	TGGAAGAGGATGTCACGCTTGGAG	GAATCAGTTCTCCCAAGAGTGTGA
	CYP4CE1	CATGATGACTAGGCGACTGCAGAC	CCATCTTGGTTATCGATTCGATGC
	CYP4DE1	CTTTCCGAATGGCTCTTCCAGTTC	CTTTCCAACTCTTCAAACGCTTGC
	CYP4DC1	GTACAACTTGTTCTCGAATGTGG	GGAGAGCAGCACATAGAGTTCAG
	CYP4DD1	CCATACGTGGCACGCACAGTTAC	TGCCTCAACACCGTCGAAATCAC
	CYP4FB1	CGTGATATGTTGGATGAGAAATCA	GATGACTCGCTCCAAATACTCCAG
	CYP4FB2	CATCCAGCTAGGCAATAGAATAC	CGCTTCATCATTGCATAATGGCC
	CYP4G115	CTGGTACGTCTACTTCCGGATGTC	CGGTGCTCTTGTCCAGGTGCACA
	CYP4G76	CTACAAACCAGATGATGTCGAGGT	CACGTCGAACTCTCTGCGAGTGGC
	CYP380C10	CAGAAATCCAGGACCGAGTATAC	CTGTACAACCGGCTGGAAGTGTGT
	CYP417A1	CTCTCATAGGCAACGCTCACCTGT	TCTGATGGAAGGTCGGATTCACCAT
	CYP417A2	GTGACTTGGTACATGAGGAGAATG	GATGCTACGAATATTCCATCAGAG
	CYP417A3	CTGATATCCAGGAGAGAGTATATC	CATGACCTGGACAATTTCTTGGAC
	CYP417B1	GATTGGTTCGACAGTGTTTCCAGC	AGACATCGGTGTACTCCAGATCTG
	CYP425A1	CTAAGACTCTATACAGCACCACAC	TCATTATCTTGATTGACATGATCC
	CYP425B1	CCTCACTCTTTGCGACTACTCACT	CGATAGCACTCCGTATCTAGATCC
	CYP426A1	GCAACTATATCTACAAGTAAACGTG	GATCTTCCTGAGGAACAAGGGAGG
	CYP439A1	CACAGATGTCTGCTTGATATTGTC	ACATCTTCTGATGTTGAGCTTGCA
	CYP439A2	CATACATCGATGCATGCTCCATGTC	CATTGATTAGTTCTAATCCAAAGTC
	CYP439B1	GTCACAATGAGCTCATTGCAACTG	GGTATTGTTGAACACAATGTTTGG
CYP3 Clade	*CYP6AX1*	CGCTTCAAGGTGAGGACTTTCAT	GTGTTGGCTGCTAGTAGTTTATCG
	CYP6AY1	GCTGTTTCACTTCTTGAGACTCCG	GCTTGAGCTGCTATAACACTCTCTG
	CYP6BD12	CATCCAGCACTCTGCTCACATTCGC	CACACCCTGTCTATCCTGGCCAC
	CYP6CS1	TCAAGAGACGCTGAGGAAGTACC	CTTCACCAAACGGTAGGTAGCTGA
	CYP6CW1	CCTTCCTACCACCAGGATCGATC	CCGTTAGACTGGCCAGGCTGCTCC
	CYP6ER1	TGGCTGTTAATCAAGAGATGCAGC	CTGAAGCGCATAGACCGGAATC
	CYP6FK2	TTGGTTCGGTCAGTAGACTCGCGA	GGCTTCTCCAGTAGTCTCCTTCG
	CYP6FL3	ATGCTGTGGTCCAAGATATCTATG	GTATCTCAGACCTCTCCAGCGG
	CYP6FL4	GATAAGGAAGGGAGACGAGATAGT	ACACTTTCACTGATAGGATGGCGA
	CYP6FU1	GGTAACCTGAGAATCTCCGATCTG	GCGGACCATCCAAATGGATGAGAT
	CYP3115A1	CCTGTGTATCAACCAAGTTCAACC	CGTGAGCTTGTGCCTCACATACC
	CYP408A1	CAGTGCATCACCTTGAAGAGTTC	CCGCTCACGAATGGTGACGATAC

	Gene name	Forward primer (5′-3′)	Reverse primer (5′-3′)
	CYP418A1	CCATTCAGCAGGACATACCACAAC	CACCATCAAATCCAAACCATAGC
	CYP427A1	CCAGTATCCAGACGTACAAAGTAG	GATCAGCGTAGATGTCACCATCC
Mito Clade	*CYP301A1*	ATACTGCTGAGCGATCCTGATCCA	GTACTTCCTTGATAAACGACCTA
	CYP302A1	GATCACAGATGAGTTTCTGGAAG	GCCACAGAATGTGATTCAGGTTG
	CYP301B1	GTACCCAGTTGTAATAGGCAATG	CGAATCGCCTGCCGAGACACATG
	CYP314A1	CTCAGCTGGTGGATACTGCATTGTC	CTGAGGGGATCACAGACATCAGC
	CYP315A1	CGATCACTTTCCGATGACGCAATC	CTTCACTCATTCGTCTGCCAATAC
	CYP353D1	CCATTCTTAGTGGATATCTCGTTC	CGGGACAGACTCTGGCTCCGAAC
	CYP404B2	CACGAGGTCCTCCAGCAATTGTAC	CCGACCTTAGTCTATACCATTC
	CYP419A1	TGGGTGATGACAGCGAATGAAGTG	AGCTGTTTCTGCAACCAATCTCG
CYP2 Clade	*CYP15G1*	GATCGACTCAGTCTGTAGAGGACG	GTACTTGTTGTCCATGTGAATGCTG
	CYP18A1	GGTTGCCAAAACTGGAGGACAGAC	CAGACACATTCTACGGCCAACTCCG
	CYP303A1	CAACACTCTGTGGACCATGCTGGC	CCATCAAATCCCTAGGCGAATCC
	CYP304H1	CTTGGATATGCTGTTTGCTGCCTC	CATCTCTGTGTGCACTGTACAAGC
	CYP305A15	CTATCCACCAGGTCCGAAGTGGC	CCTTTCTCCCATTGAGCGGAGAC
	CYP306A2	GTGCACATGGATCCCCAAATTTGG	CTTCAGGTATTGCATCATCTGGCAA
	CYP307A2	CAGCAGCCGAATACTGGGAAGCG	CTGGCCTCGTGTCAATCAGGTCC
	CYP307B1	GTGTCATTGCTGGTTATACAGTAG	GGTGATAGGCTAAACCTGTGCAG
Reference gene	guanine-N (7)-methyltransferase gene (*Nl18S*)	GTAACCCGCTGAACCTCCT	TCCGAAGACCTCACTAAATC
dsRNA	*CYP6ER1*-RNAi	taatacgactcactatagg GTCAACTTCTACGTTTACTCCTATTG	taatacgactcactatagg ATCACATTCAGCCCGTAGTTGTTTG
	GFP-RNAi	taatacgactcactatagg AAGGGCGAGGAGCTGTTCACGG	taatacgactcactatagg CAGCAGGACCATGTGATCGCGC

Supplementary Table S2. Validation of the modeled structures of CYP6ER1

Protein Name	Template	Sequence Identity	Coverage	Verify 3D	ERRAT	G-Factors		
						Dihedrals	Covalent	Overall
CYP6ER1	3nxu.2.A	33.50%	0.85	87.44%	85.40	-0.18	-0.08	-0.12
